# AIDS Vaccine for Asia Network (AVAN): Expanding the Regional Role in Developing HIV Vaccines

**DOI:** 10.1371/journal.pmed.1000331

**Published:** 2010-09-21

**Authors:** Stephen J. Kent, David A. Cooper, Mean Chhi Vun, Yiming Shao, Linqi Zhang, Nirmal Ganguly, Budiman Bela, Hiko Tamashiro, Rossana Ditangco, Supachai Rerks-Ngarm, Punnee Pitisuttithum, Nguyen Van Kinh, Alan Bernstein, Saladin Osmanov, for the AIDS Vaccine for Asia Network investigators and supporters

**Affiliations:** 1 University of Melbourne, Parkville, Victoria, Australia; 2 University of New South Wales, Darlinghurst, New South Wales, Australia; 3 National Center for HIV/AIDS, Dermatology and STIs (NCHADS), Phnom Penh, Cambodia; 4 State Key Laboratory for Infectious Disease Control and Prevention, National Center for AIDS/STD Control and Prevention, Beijing, China; 5 Tsinghua University, Chinese Academy of Medical Sciences and Peking Union Medical College, Beijing, China; 6 Indian Council of Medical Research, New Delhi, India; 7 University of Indonesia, Jakarta, Indonesia; 8 Hokkaido University, Hokkaido, Japan; 9 Research Institute for Tropical Medicine, Manila, Philippines; 10 Department of Disease Control, Ministry of Public Health, Bangkok, Thailand; 11 Faculty of Tropical Medicine, Mahidol University, Bangkok, Thailand; 12 National Institute of Infectious and Tropical Diseases (NIITD), Hanoi, Viet Nam; 13 Global HIV Vaccine Enterprise, New York, New York, United States of America; 14 World Health Organization/Joint United Nations Programme on HIV/AIDS, Geneva, Switzerland

## Abstract

Yiming Shao and colleagues describe the work of AVAN, the AIDS Vaccine for Asia Network, which aims to strengthen its regional efforts in finding an AIDS vaccine.

**Summary Points**

- The HIV/AIDS pandemic continues to spread and an AIDS vaccine is urgently needed.
- Regional alliances and international collaborations can foster the development and evaluation of the next generation of AIDS vaccine candidates.
- The importance of coordinating and harmonizing efforts across regional alliances has become abundantly clear.
- We recently formed the AIDS Vaccine for Asia Network (AVAN) to help facilitate the development of a regional AIDS vaccine strategy that accelerates research and development of an AIDS vaccine through government advocacy, improved coordination, and harmonization of research; develops clinical trial and manufacturing capacity; supports ethical and regulatory frameworks; and ensures community participation.

## The Challenge

The HIV/AIDS pandemic continues to spread and an AIDS vaccine is urgently needed. While facing unprecedented challenges, AIDS vaccine development activities are continuing around the globe [1],[2]. Recent results of the Thai Phase III vaccine trial are renewing such efforts [3]. In accordance with the goals of the Global HIV Vaccine Enterprise (the Enterprise) [4], there is now clear recognition of the role that regional alliances can play in fostering and facilitating AIDS vaccine development [5], and there is broad agreement that international collaborations are the most effective way forward to develop and evaluate the next generation of AIDS vaccine candidates [6].

In response to these challenges, the Asian region has recently formed the AIDS Vaccine for Asia Network (AVAN), with a clear vision and mission (Box 1).

**Box 1. AVAN's Vision and Mission**

Vision

- To develop a safe and effective AIDS vaccine and ensure its access as a part of a comprehensive public health strategy for the control of new HIV infections across the Asian region.

Goals

- To accelerate the development of an AIDS vaccine through expanding capacity for all aspects of AIDS vaccine research and development.
- To build up regional resource centres and collaborative platforms to promote innovative AIDS vaccine research strategies.
- To enlarge the pipeline of candidate AIDS vaccines suitable for use among Asian populations.
- To strengthen capacity and harmonize regulatory and ethical frameworks for the conduct of clinical trials that comply with internationally recognized standards.
- To actively involve community partners at all stages of AIDS vaccine development, clinical trials, and future use.
- To promote and share manufacturing and production capacity in the region, in compliance with GMP standards, both for clinical trials and for the potential of high demand for an AIDS vaccine in Asia.
- To engage with and advocate to governments and the private sector to commit to and provide political and financial support for all phases of AIDS vaccine research and development, including licensing and future access.
- To facilitate collaboration between Asian scientists and industry with the rest of the world.
- To align with the values and strategic goals of the Global HIV Vaccine Enterprise.

## The Need for AVAN: The HIV Epidemic in the Asian Region

The total Asia population is approximately 4 billion people—just over 60% of the world population. More than 500 million people are considered to be at-risk for HIV exposure and infection, including youth, injecting drug users (IDUs), sex workers, men who have sex with men (MSM), and mobile populations. Close to 5 million people have already been infected with HIV across the region, although the epidemic is highly variable across different countries, communities and populations [7].

Asian HIV sub-epidemics are driven primarily by unsafe sex and drug injection and are therefore currently concentrated in groups with higher risk for HIV; however, the epidemic is slowly spreading into the general population. Although impressive results have been achieved following the scaling up of available HIV prevention strategies in some countries [8], the level of prevention, care, and treatment coverage among risk-associated groups remains unacceptably low.

**Figure 1:**
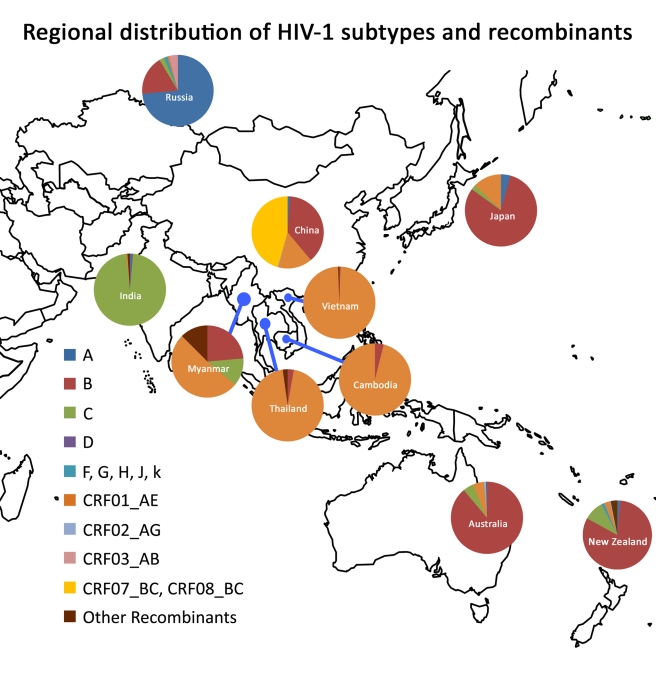
Regional distribution of HIV-1 subtypes and recombinants. The relative proportion of each subtype/recombinant is shown in the pie chart for each country. The data on Figure 1 are from the former studies in the region and estimates made by AVAN Task Force members of the manuscript based on the unpublished data of their own research projects.

The main factors influencing HIV vaccine development in the region include circulation of multiple HIV subtypes and circulating recombinant forms (CRFs) transmitted through the various modes of transmission, diverse host genetics, and disparate social, cultural, and political contexts in the region. While IDUs were the initial driving force of many HIV epidemics in Asian countries, sexual transmission has gradually taken over. Significant increases in the number of new HIV infections are also expected to occur across the region among MSM. The geographic distribution of HIV subtypes and CRFs is relatively homogeneous but varies by subcontinent: CRF_07B′/C, B′, and CRF01_AE in China [9]–[11]; subtype C in India [12]; and CRF01_AE in Thailand [13] (Figure 1). Unfortunately, HIV incidence data remain incomplete and need to be bolstered for optimal planning of vaccine studies. These interrelated factors may affect AIDS vaccine research and development in the region and therefore should be given due consideration in planning future studies. The complexity of the epidemic in the region poses substantial challenges but also affords tremendous opportunities to accelerate AIDS vaccine development considering the relatively good infrastructure of doing epidemiology study in the region.

## Current Efforts in HIV Vaccine Research and Development in Asia

Significant basic and clinical HIV vaccine research and development efforts are already underway across the region, with notable achievements (see Table 1). Several Asian countries now have National AIDS Vaccine Plans and Strategies. Further harmonization and consolidation of efforts will result in more productive associations and acceleration of AIDS vaccine development.

**Table 1:** AIDS Vaccine Trials across Asia to Date.

Country	Vaccine	Sponsor	Subtype	Phase/Time	Reference
Thailand	V3 peptides	UBI	Multiclade	I/1994	[32]
	gp120	Vaxgen, Chiron	E, B, B′/E	I/II,III/1995	[33]
	gp160	DoD	E	I/II	
	Canarypox+gp 160 vs. gp 120	US NIH/DoD	B/E	I/II/2003	[34]
	Adenovirus type 5	Merck	B	I/II/2003	
	MVA	US NIH/DoD	A/E	I	[35]
	DNA+fowlpox	Australia	A/E	I/2004	[36]
	Canarypox+gp 120	US NIH/DoD	B/E	III/2006	[3]
China	V3 peptides	UBI Co.	Multiclade	I/1993	[31]
	DNA+MVA	Baike Co.	CRF08_B/C	I/2005	
	Tiantan vaccinia replicative	China CDC/EU	CRF 07_B/C	I/2006	[15]
	DNA+Tiantan replicative	China CDC	CRF 07_B/C	I/2008	[37]
	DNA+MVA	Baike Co.	CRF08_B/C	II/2009	
India	Adeno-associated virus	IAVI	C	I/2003	[18]
	MVA	IAVI	C	I/2005	[20]
	AAV vs. DNA+MVA	IAVI	C	I/2009	[38]
Australia	DNA+fowlpox	US NIH	B	I/2004	[21]
	DNA+fowlpox	Australia	A/E	I	[36]

CDC, Center for Disease Control and Prevention; DoD, Department of Defense; EU, European Union.

### Thailand

AIDS vaccine activities have a long-standing history in Thailand, beginning in the mid-1990s [14], reflecting a strong political will to invest in AIDS vaccine research and development to stem the epidemic in Thailand. Thailand has conducted Phase I, II, and III trials with the AIDSVax gp120 B′/E vaccine, as well as studies of other candidate vaccines (Table 1).

Most notably, Phase III trials of the ALVAC (Canarypox, vCP1521) and AIDSVAX gp120 B/E prime-boost regimens have also been completed. This landmark trial, the largest AIDS vaccine efficacy trial conducted to date involving over 16,000 volunteers, showed a modest (31%) but significant reduction in new HIV infections and is the first time that an AIDS vaccine has exhibited protection against HIV acquisition [3].

The Thai experience in successfully conducting two Phase III efficacy trials has provided world-class expertise in collaborative teamwork, community engagement activities, good clinical practice, ethical compliance, sample repositories, immunology studies, good laboratory practices, volunteer retention strategies, and large-scale data management approaches. These efforts and investments resulted in an extraordinary volunteer retention rate of 96% during the latest Phase III trial. The Thai experience offers a good example of what can be achieved through international collaboration. In addition, several preclinical studies with promising HIV vaccine candidates are now ongoing in Thailand. Researchers in Chulalongkorn University are working on a HIV-1 AE/B mosaic DNA vaccine as well as techniques to improve vaccine delivery system. The Thailand Regional Laboratories in the Comprehensive Antibody-Vaccine Immune Monitoring Consortium are working with researchers from the Los Alamos National Library and the National Cancer Institute on a cocktail of the mosaic DNA vaccine and DNA/vaccinia primed-boosted strategy. These candidates were tested in mice and have shown a promising immunogenicity result.

### China

The Chinese government has significantly invested in AIDS vaccine research. China's vaccine scientists have recently formed the Chinese AIDS Vaccine Initiative (CAVI), supported by the government of China. The initiative includes projects aimed at cohort development, vaccine vector design, establishment of clinical trial units, a manufacturing facility using principles of good manufacturing practice (GMP), a primate centre, a humanized mouse centre, and technical platforms encompassing both neutralizing antibody and T cell expertise.

Clinical studies conducted so far include three Phase I trials: V3 peptide vaccine in 1993, DNA/MVA (modified vaccinia Ankara) in 2005, and a DNA/Tiantan vaccinia strain (replication-competent) in 2007 (Table 1). Pre-clinical studies with DNA and modified Tiantan vaccinia, as well as new versions of DNA, vaccinia, and adenovirus vector approaches for inducing mucosal immunity show considerable promise [15]–[17]. China's AIDS vaccine programme now has substantial capacity in primate centres, vaccine production, and clinical trial sites development that will all help to facilitate further research and development in the region.

### India

AIDS vaccine trials have accelerated significantly in India in recent years. Activities include an adeno-associated virus vector Phase I trial, modified MVA vector vaccine trials [18],[19], and a recent DNA/MVA Phase I prime-boost trial initiated in 2009 [20]. The International AIDS Vaccine Initiative (IAVI) is supporting Indian AIDS vaccine trials and applied research on neutralizing antibody immunogens [21]. Exploration of several novel concepts, including prime-boost regimens with CD40L adjuvants, as well as research towards development of improved Env-based immunogens, is also progressing. Improved awareness of AIDS vaccine issues, training of staff, and development of trial sites have substantially improved the capacity of conducting AIDS vaccine research and clinical trials within India.

### Australia

Australia has long-standing capacities in both fundamental HIV research and clinical trial activities. A consortium, termed the Australia-Thai HIV Vaccine Consortium, conducted two recent trials, first in Sydney and then in Bangkok, of a DNA-prime and Fowlpox virus boost vaccine using both subtype B and CRF01_AE strains [22]. Australia was a clinical trial site of the adenovirus-based efficacy (STEP) trial that was not efficacious. Through collaborations, several new vaccine candidates are emerging. These include peptide-based vaccines, gp140 immunogens, recombinant influenza vectors, and particle-based vaccine strategies [23],[24]. In addition, improved and simpler assays to measure T cell immunity and antibody-dependent cellular cytotoxicity (ADCC) are emerging [25],[26]. These scientific approaches could be accelerated into expanded clinical trials in future collaborations across the Asian region.

### Japan

Japan has developed a pipeline of promising AIDS vaccine candidates. These include recombinant *Mycobacterium bovis*/Bacillus Calmette-Guérin (BCG) vector-based regimens that are moving towards clinical trial development in collaboration with Thai groups [27]. In addition, novel Sendai virus vectors are being developed with the opportunity to deliver vaccines mucosally to induce mucosal immunity [28]. Sendai virus vectors combined with DNA vaccine candidates are moving towards clinical trials in collaboration with IAVI.

## The Evolution, Mission, and Strategic Plan Development of AVAN

The first Asian meetings devoted to AIDS vaccines took place in Japan, China, and Thailand in the late 1990s. The initial efforts were followed by a World Health Organization (WHO)/Joint United Nations Programme on HIV/AIDS (UNAIDS) regional consultation on AIDS vaccines in Japan in 2006 and in Beijing in 2009 [29]–[31]. The challenges for engaging countries and communities in expanding pre-clinical and clinical trials, and in enhancing regulatory and manufacturing capacity to accelerate the development of AIDS vaccines in the region, led to the establishment of AVAN, announced at the 2009 AIDS Vaccine Conference in Paris. AVAN has had regular consultations towards developing an Asia-specific Strategic AIDS Vaccine Plan in alignment with the Enterprise Scientific Strategic Plan [6].

An AVAN Task Force was recently established to foster further dialogue and set the stage for the development of principles and priorities for a focussed, forward-looking and effective strategic plan for the network. Eventually, the Task Force will be replaced by a steering committee to guide implementation of a strategic plan. An initial AVAN Task Force secretariat has been established, currently based in China, with strong support from the Chinese AIDS Vaccine Initiative, WHO, the Enterprise, and the Chinese government. The Task Force and secretariat foster forums for consultation and networking around many of the most critical future issues. Regional workshops are planned to further implement the emerging strategic plan in June 2010 in Bangkok, Thailand, and in August/September 2010 in Sapporo, Japan. AVAN plans to support and promote the development of regional resource centres to provide information, a reagents repository, protocols, and training on the many aspects of AIDS vaccine research and development. Coordination and harmonization of various aspects of AIDS vaccine development, particularly regional vaccine immunology evaluation laboratories and regulatory reviews, is a high priority in moving AIDS vaccine research forward across Asia. AVAN shall ultimately become the key advocate for AIDS vaccine research in the region, building upon the trust created through working together in collaborative partnerships in the region and with support from the Enterprise.

## Past Collaborations and Future Opportunities

AVAN aims to make a significant impact as the region undertakes a stronger role in AIDS vaccine research and development. Asian investigators have substantial capacity to conduct basic research, which could be significantly enhanced through better collaboration. The current pipeline of new vaccine candidates needs further expansion with more innovative concepts and trans-national efforts to accelerate research. Clinical trial capacity could be quickly exhausted with multiple, concurrent efficacy trials in the future. As partially efficacious AIDS vaccines are identified either in Asia or around the world, determining their effectiveness in regional epidemics involving differing HIV virus subtypes will be critical. Considering the large number of IDUs in many countries of the region still with a high incidence of HIV infection, Asia may serve as the ideal site suitable for testing a vaccine to prevent parental HIV transmission.

Building upon and maintaining the substantial efficacy trials expertise in Thailand is both an opportunity and a challenge. Substantial opportunities exist to harmonize the regulatory and ethical review of AIDS vaccine trials across the region—too often, delays experienced in initiating trials with vectors of known safety profile are very lengthy. Data management and data sharing across the region can also be enhanced. Opportunities for training young immunologists and virologists in the region to foster the next generation of scientists need to be encouraged and supported. Technology transfer from Western countries to Asian countries also needs to be strengthened. Finally, tapping into the large cost-effective vaccine manufacturing capacity in several Asian countries is an opportunity and priority.

International groups have previously provided high level support for AIDS vaccine activities in the Asian region, including research and technical transfer, funding and policy. In the past two decades, the US National Institutes of Health (NIH) has provided extensive support for basic and clinical research across many Asian countries and funded the majority of the RV 144 trial. The Walter Reed Army Institute of Research and the US Military HIV Research Program (MHRP), in collaboration with the Royal Thai Army, sponsored several clinical trials in Thailand, including the RV144 trial. IAVI is supporting clinical trial capacity building along with policy and preparedness activities. The EuroVacc AIDS Vaccine network has included the Chinese HIV strains in their development work in view of future plans of conducting trials in the region. The Collaboration for AIDS Vaccine Discovery (CAVD), supported by the Bill & Melinda Gates Foundation, is also looking towards enhancing research on AIDS vaccines in Asia in coming years. WHO-UNAIDS and the Global HIV Vaccine Enterprise are actively engaging in policy development, assisting the development of national AIDS vaccine plans, and providing a global umbrella for AIDS vaccine development activities in Asia. Further investment in AIDS vaccine development by the most developed nations in the Asian region would be welcome. The consolidation of this collaborative support will be critical in progressing future vaccine development efforts.

## Conclusions

The development of an effective AIDS vaccine has never been more urgent, particularly for the hundreds of millions of people across Asia at substantial risk of acquiring HIV. Similarly, the necessity of coordinating and harmonizing efforts across regional alliances has become abundantly clear. AVAN has been initiated to meet these needs and actively facilitate the development of a regional AIDS vaccine strategy that accelerates research and development of an AIDS vaccine through government advocacy, improved coordination and harmonization of research; develops clinical trial and manufacturing capacity; supports ethical and regulatory frameworks; and ensures community participation.

## Abbreviations

ADCC: antibody-dependent cellular cytotoxicity
AVAN: AIDS Vaccine for Asia Network
BCG: *Mycobacterium bovis*/Bacillus Calmette-Guérin
CAVD: Collaboration for AIDS Vaccine Discovery
CAVI: Chinese AIDS Vaccine Initiative
CDC: Center for Disease Control and Prevention
CRF: circulating recombinant form
DoD: Department of Defense
the Enterprise: Global HIV Vaccine Enterprise
EU: European Union
GMP: good manufacturing practice
IAVI: International AIDS Vaccine Initiative
IDU: injecting drug user
MHRP: US Military HIV Research Program
MSM: men who have sex with men
MVA: modified vaccinia Ankara
NIH: National Institutes of Health
UNAIDS: World Health Organization (WHO)/Joint United Nations Programme on HIV/AIDS
